# Regional Disparities in Educational Attainment and Its Impact on Cognitive Health in Western and Eastern Africa

**DOI:** 10.1002/alz70860_107528

**Published:** 2025-12-23

**Authors:** Carolina Scaramutti, Dingtian Cai, Farid Rajabli, Larry D Adams, Kara L Hamilton‐Nelson, Michael L Cuccaro, Daniel A. Dorfsman, Joshua O Akinyemi, Kyle M Scott, Motunrayo Coker, Kazeem Akinwande, Samuel Diala, Patrice G Whitehead, Mayowa Ogunronbi, Albertino Damasceno, Andrew F Zaman, Yared Z Zewde, Biniyam A Ayele, Allison M Caban‐Holt, David Ndetei, Anthony J Griswold, Fred Stephen Sarfo, Susan H. Blanton, Albert Akpalu, Kolawole Wahab, Katalina F. McInerney, Reginald Obiako, Olusegun Baiyewu, Pedro R Mena, Njideka U Okubadejo, Mayowa O Owolabi, Izri M Martinez, Brian W Kunkle, Jeffery M Vance, Christiane Reitz, Giuseppe Tosto, William S Bush, Scott M Williams, Raj Kalaria, Jonathan L Haines, Adesola Ogunniyi, Goldie S Byrd, African Dementia Consortium, Alzheimer's Disease Sequencing Project (ADSP), Rufus O Akinyemi, Margaret Pericak‐Vance, Azizi A Seixas

**Affiliations:** ^1^ Department of Psychiatry and Behavioral Sciences, University of Miami Miller School of Medicine, Miami, FL, USA; ^2^ John P. Hussman Institute for Human Genomics, University of Miami Miller School of Medicine, Miami, FL, USA; ^3^ University of Miami, Miami, FL, USA; ^4^ Dr. John T. Macdonald Foundation Department of Human Genetics, University of Miami Miller School of Medicine, Miami, FL, USA; ^5^ University of Miami Miller School of Medicine, Miami, FL, USA; ^6^ College of Medicine, University of Ibadan, Ibadan, Oyo, Nigeria; ^7^ Neuroscience And Ageing Research Unit, Institute for Advanced Medical Research and Training, College of Medicine, University of Ibadan, Ibadan, Nigeria; ^8^ Cell Biology and Genetics Unit, Department of Zoology, University of Ibadan, Ibadan, Nigeria; ^9^ Federal Medical Centre, Abeokuta, Ogun State, Nigeria; ^10^ Faculty of Medicine, Eduardo Mondlane University, Maputo, Mozambique; ^11^ College of Health Sciences, Addis Ababa University, Addis Ababa, Ethiopia; ^12^ Global Brain Health Institute, San Francisco, CA, USA; ^13^ Wake Forest University School of Medicine, Winston‐Salem, NC, USA; ^14^ Maya Angelou Center for Health Equity, Wake Forest School of Medicine, Winston‐Salem, NC, USA; ^15^ Africa Institute of Mental and Brain Health, Nairobi, Kenya; ^16^ Africa Mental Health Research and Training Foundation, Nairobi, Kenya; ^17^ University of Nairobi, Nairobi, Kenya; ^18^ Komfo Anokye Teaching Hospital, Kumasi, Ghana; ^19^ Kwame Nkrumah University of Science and Technology, Kumasi, Ghana, Kumasi, Ghana; ^20^ University of Ghana Medical School, Accra, Ghana; ^21^ Department of Medicine, University of Ilorin, Ilorin, Nigeria; ^22^ Division of Neurology, Department of Medicine, Ilorin, Nigeria; ^23^ Department of Neurology, University of Miami Miller School of Medicine, Miami, FL, USA; ^24^ Neurology Unit, Department of Medicine, Ahmadu Bello University/Ahmadu Bello University Teaching Hospital, Zaria, Nigeria; ^25^ Ahmadu Bello University, Zaria, Kaduna, Nigeria; ^26^ African Dementia Consortium, Lagos, Nigeria; ^27^ College of Medicine, University of Lagos, Lagos, Nigeria; ^28^ Institute for Advanced Medical Research and Training, College of Medicine, University of Ibadan, Ibadan, Oyo, Nigeria; ^29^ John P. Hussman Institute for Human Genomics, Miller School of Medicine, University of Miami, Miami, FL, USA; ^30^ University of Miami, Miller School of Medicine, John P. Hussman Institute for Human Genomics, Miami, FL, USA; ^31^ Columbia University, New York, NY, USA; ^32^ Columbia University Medical Center, New York, NY, USA; ^33^ Gertrude H. Sergievsky Center, Taub Institute for Research on the Aging Brain, Departments of Neurology, Psychiatry, and Epidemiology, College of Physicians and Surgeons, Columbia University, New York, NY, USA; ^34^ Columbia University Irving Medical Center, New York, NY, USA; ^35^ Department of Neurology, College of Physicians and Surgeons, Columbia University, and the New York Presbyterian Hospital, New York, NY, USA; ^36^ Department of Population and Quantitative Health Sciences, Institute for Computational Biology, Case Western Reserve University, Cleveland, OH, USA; ^37^ Case Western Reserve University, Cleveland, OH, USA; ^38^ Case Western Reserve University School of Medicine, Cleveland, OH, USA; ^39^ Institute for Ageing and Health, Newcastle University, Newcastle, United Kingdom; ^40^ Neuroscience and Aging Research Unit, Institute of Advanced Medical Research and Training, College of Medicine, University of Ibadan, Ibadan, Nigeria, Ibadan, Oyo, Nigeria; ^41^ Perelman School of Medicine, University of Pennsylvania, Philadelphia, PA, USA; ^42^ University of Pennsylvania, Philadelphia, PA, USA; ^43^ John P. Hussman Institute for Human Genomics, Miller School of Medicine, Miami, FL, USA

## Abstract

**Background:**

Education is a crucial social determinant of health, influencing opportunities, socioeconomic status, and health outcomes. Regional disparities in educational attainment and their effects on cognitive health are especially notable in low‐ and middle‐income countries (LMICs), where access to education and healthcare varies widely. This study explores the role of education in health disparities, focusing on two distinct African regions—Western and Eastern Africa—using data from the READD‐ADSP DAWN Study.

**Method:**

This cross‐sectional study analyzed data from Western Africa (*n* = 756) and Eastern Africa (*n* = 689). Variables included early‐life learning disability, special education participation, educational attainment (None, Elementary, High School, College, Graduate/Professional), and cognitive health diagnoses (Non‐Cognitively Impaired, MCI, AD, Dementia, Other or No Diagnosis). A Wilcoxon rank sum test assessed differences in educational attainment between cognitive diagnostic groups. Logistic regression with a logit link function examined the relationship between years of education and the likelihood of a primary diagnosis of Alzheimer's Disease or Dementia.

**Result:**

In the Eastern region sample, the Wilcoxon rank sum test revealed a significant difference in education levels between individuals with and without a cognitive health diagnosis (W=68233, *p* <0.001). Logistic regression showed that education was a significant predictor of diagnosis (β=−0.04891, SE=0.01641, z=−2.980, *p* = 0.00288). Higher education levels were associated with lower odds of a cognitive health diagnosis. The model fit was reasonable, with a residual deviance of 927.65 and an AIC of 931.65. In the Western region sample, the Wilcoxon rank sum test also showed a significant difference in education levels between individuals with and without a cognitive health diagnosis (W=81364, *p* = 0.02669). Logistic regression confirmed that years of education significantly predicted diagnosis status (β=−0.02640, SE=0.01076, z=−2.454, *p* = 0.0141), with higher education linked to lower odds of being diagnosed with a cognitive health diagnosis. The model fit was adequate, with a residual deviance of 1060.2 and an AIC of 1064.2.

**Conclusion:**

These findings further support the protective role of education against cognitive decline in both regions.

